# Prevalence of psychiatric disorders among the adult population in a rural community of Jammu, India: a cross-sectional study

**DOI:** 10.3389/fpsyt.2024.1433948

**Published:** 2024-10-25

**Authors:** Sandeepa Bailam, Amrit Sudershan, Sheetal, Mohd Younis, Manu Arora, Hardeep Kumar, Parvinder Kumar, Dinesh Kumar

**Affiliations:** ^1^ Department of Community Medicine, Government Medical College, Jammu, Jammu and Kashmir, India; ^2^ Department of Community Medicine, Acharya Shri Chander College of Medical Sciences and Hospital, Jammu, Jammu and Kashmir, India; ^3^ Institute of Human Genetics, University of Jammu, Jammu, Jammu and Kashmir, India; ^4^ Department of Human Genetics, Sri Pratap College, Cluster University Srinagar, Kashmir, Jammu and Kashmir, India; ^5^ Department of Psychology, Govt., Degree College, Billawar, Kathua, Jammu and Kashmir, India; ^6^ Department of Psychology, University of Jammu, Jammu, Jammu and Kashmir, India; ^7^ Department of Zoology, University of Jammu, Jammu, Jammu and Kashmir, India; ^8^ Department of Psychiatry, Government Medical College, Jammu, Jammu and Kashmir, India; ^9^ Department of Neurology, Super Specialty Hospital, Government Medical College, Jammu, Jammu and Kashmir, India; ^10^ Department of Community Medicine, All India Institute of Medical Science, Jammu, Jammu and Kashmir, India

**Keywords:** psychiatric disorders, rural areas, Jammu, socio-demographic factors, mental health disparities

## Abstract

**Background:**

Mental health is crucial for overall well-being, but rural areas often face difficulties in accessing mental health services and understanding psychiatric disorders.

**Aim:**

This study aimed to address these issues by assessing the prevalence of psychiatric disorders among adults in rural Jammu and examining how socio-cultural and demographic factors are related to these disorders.

**Methods:**

A sample of 1,016 adults from rural Jammu was surveyed using the Mini International Neuropsychiatric Interview (MINI). Data were collected through house-to-house visits conducted by trained investigators. Descriptive and inferential statistics, including frequency distribution and odds ratio, were employed to analyze the data respectively.

**Results:**

Overall, 20.67% of the participants had a psychiatric disorder. Alcohol dependence was the most common condition, affecting 12.30% of the individuals, followed by generalized anxiety disorder at 3.14%. The study found significant associations between psychiatric disorders and several factors. Specifically, older people were more likely to have psychiatric disorders, with an odds ratio of 3.7 [2.07-6.59]. Married individuals also had a higher likelihood of experiencing psychiatric disorders (OR: 2.3 [1.55-3.54]). Those with less schooling were at an increased risk, with an odds ratio of 7.77 [2.31-26.09], and people from lower socioeconomic backgrounds were more likely to have these disorders as well (OR: 5.1 [2.4-10.5]).

**Discussion and conclusion:**

The findings underscore the complex association between socio-demographic factors and mental health outcomes in rural areas of Jammu region. Addressing these disparities requires targeted interventions and policies that account for the unique socio-cultural contexts of rural populations. By understanding the specific challenges faced by these communities, policymakers and healthcare providers can develop more effective strategies to enhance mental health services and promote well-being.

## Introduction

Health is considered the most valuable asset, encompassing physical, mental, and social well-being, and not merely the absence of disease or infirmity, as defined by the World Health Organization (WHO) (1). An important implication of this definition is that mental health is more than just the absence of mental disorders or disabilities; it also includes resilience, social functioning, and overall quality of life. Recent advancements in psychiatry have significantly deepened our understanding of mental illnesses and their complexities. This growing knowledge has led to new perspectives on the causes of these conditions and the development of more effective treatment strategies (2). In light of this, there is increasing acknowledgment of the necessity to comprehend the prevalence and factors influencing psychiatric disorders, especially in rural and underserved communities (3) such as rural areas of Jammu (R.S. Pura). Numerous neurological diseases affecting the Jammu area have been the subject of notable research, including headaches (4), epilepsy (5, 6), and others (7, 8). Likewise, the Kashmir region of Jammu and Kashmir, India (UT) has been the site of many studies about different types of mental diseases such as depression, post-traumatic stress disorders (PTSD), and generalized anxiety disorders (9–12).

However, mental health research specifically in rural Jammu, such as R.S. Pura, is noticeably lacking. This may be due to the region’s unique socio-political challenges, as it is located near the international border between India and Pakistan. The limited research in this area underscores its status as an underserved community, warranting a focused investigation into mental health challenges. Therefore, this study primarily aims to determine the frequency of psychiatric disorders among people residing in rural Jammu. Additionally, we hope to shed light on this crucial aspect of mental health in the area by exploring the link between demographics, culture, socioeconomic status, and mental illness.

## Method

### Ethical consideration

Ethical clearance was secured from the Institutional Ethical Committee (IEC) of Government Medical College, Jammu, Jammu and Kashmir. All participants were asked to provide their informed consent before they were involved. They understood that their participation in the study would remain anonymous and confidential.

### Sample size

A sample size of 1016 participants was determined to estimate mental disorder prevalence with a 10% baseline within a 20% margin of error. This estimation was conducted at a significance level of 5% (95% confidence level) and a power of 80%. Accounting for a 10% non-response rate ensures robust analysis and meaningful interpretation of the study findings.

### Participants, sampling method, and data collection

The area under study, named R.S. Pura, is located in the Jammu district at 32.6049° N, 74.7315° E, close to the international border between India and Pakistan. It covers a total area of 245 km², with 238.48 km² being rural and 6.78 km² being urban. Employing a simple random sampling method, 43 villages within the Miran Sahib block of R.S. Pura (rural area) were initially divided into 4 different zones (excluding urban areas) based on the criteria of “geographical proximity.” One zone was then randomly selected using simple random sampling. Within the chosen zone, a list of villages was compiled, and four villages—Benagarh, Ullawalchak, Ghazia, and Kotlimianfateh—were randomly selected for inclusion in the study. To ensure the representativeness of the sample, we excluded villages where the population had resided for less than 5 years and those with predominantly urban features. These features included characteristics such as significant infrastructure (e.g., large commercial centers, extensive transportation networks) and a predominance of non-agricultural occupations. These criteria were evaluated through preliminary surveys and local knowledge to ensure that only rural villages, which align with the study’s focus, were included. These criteria were evaluated through preliminary surveys and local knowledge to ensure that only rural villages, which align with the study’s focus, were included^1^.

After selecting the villages, we contacted opinion leaders, including the Sarpanch (the elected head of the village) and other key individuals, to brief them about the study’s purpose. They were asked to disseminate information about the upcoming study during village meetings to foster cooperation.

Before data collection began, the investigators underwent two weeks of training led by a consultant psychiatrist (M.A.). With this preparation complete, house-to-house visits commenced, during which trained investigators obtained consent from eligible participants before collecting data. All individuals in a given household who met the inclusion and exclusion criteria were invited to participate in the study. Participants were briefed about the study before data collection. Over the course of one year, relevant demographic information was recorded. All adult participants (aged 18 to 60 years) were personally interviewed by the investigators (S.B., M.A., D.K., and team) using the MINI tool (13) and a custom sociodemographic form. The demographic information included age group, socio-economic status, social caste, marital status, and educational status.

**Figure 1:**
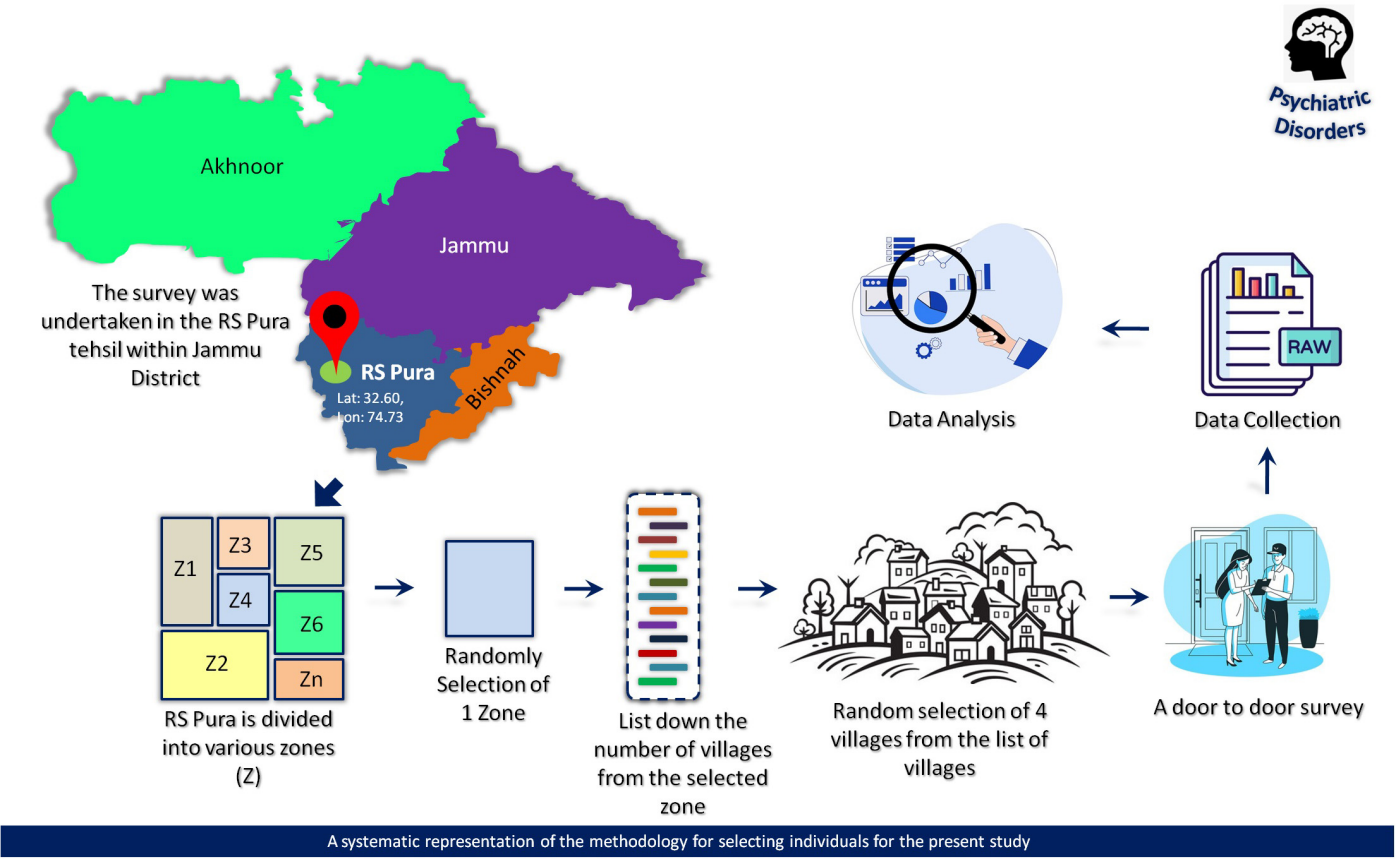
Systematic representation of the recruitment of individuals for data collection.

For cases where initial screening results were ambiguous or unclear, final diagnoses were determined by psychiatrists (M.A.). During the screening process, individuals who initially reported symptoms of psychiatric disorders, referred to as ‘positive respondents,’ were further assessed using the MINI to evaluate and diagnose potential conditions. Throughout the recruitment process, the inclusion and exclusion criteria were consistently applied. Inclusion criteria include years of age equal to or higher than 18, and less than or equal to 60 years, residing in the region for at least 5 years. Exclusion criteria: severe cognitive impairment or inability to communicate effectively. Additionally, it is important to note that if a household was locked during the initial door-to-door visit and remained locked on a second visit, that household was excluded from the sample. Confidentiality and privacy were ensured during data collection. A detailed flow diagram illustrating the recruitment of samples from the population is provided in **Figure 1**.

### Statistical analysis

The data was first entered into Microsoft Excel to organize and analyze the information. We began by providing a clear summary of the participant’s demographic characteristics and the prevalence of psychiatric disorders. To achieve this, we used descriptive statistics, specifically frequency distribution, to present the discrete variables in an easily understandable format i.e., percentage (%). For the inferential statistics, we applied a logistic regression model, which allowed us to calculate the odds ratios (2 x 2 table). This helped us explore the association between socio-cultural and demographic factors and the prevalence of psychiatric disorders. We set a significance level of p < 0.05, meaning that any results with a p-value less than 0.05 were considered statistically significant. Data analysis was done in Medcalc (MedCalc Statistical Software version 19.2.6 (MedCalc Software bv, Ostend, Belgium; https://www.medcalc.org; 2020), and SPSS version 17(IBM Corp. Released 2017. IBM SPSS Statistics for Windows, Version 25.0. Armonk, NY: IBM Corp.).

## Result

**Figure 2:**
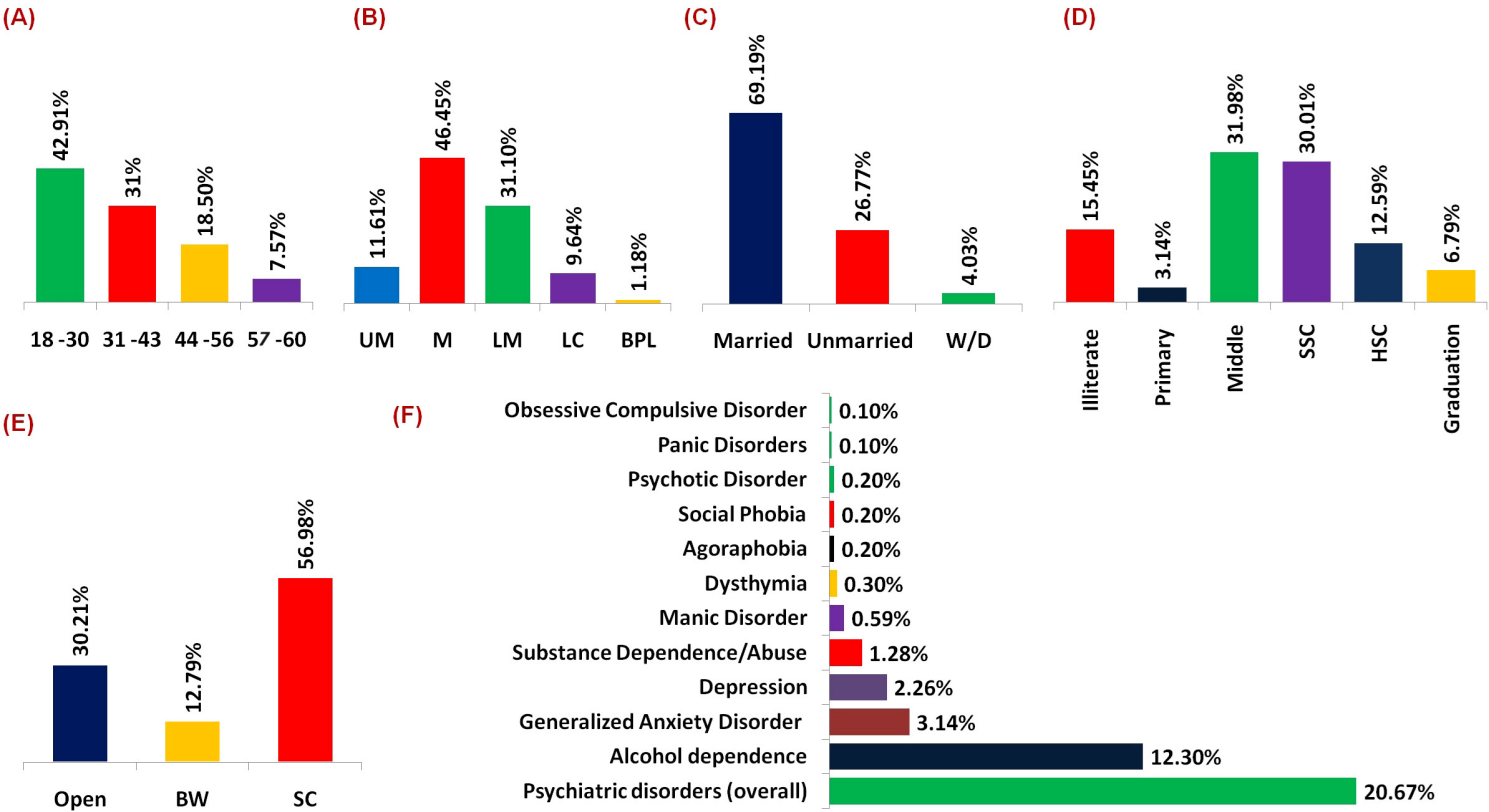
**(A)** Bar graph illustrating the distribution of individuals across various age groups, highlighting a predominant presence within the 18-30 years cohort. **(B)** Bar graph delineating the distribution of individuals across distinct socio-economic strata, with a notable concentration within the middle-class segment [UM, Upper Middle; M, Middle; LM, Lower Middle; LC, Lower Class; BPL, Below Poverty Line]. **(C)** Bar graph portraying the distribution of individuals based on marital status [Widow/Divorce]. **(D)** Bar graph depicting the educational attainment levels of the surveyed populace [SSC, Secondary School; HSC, Higher Secondary School]. **(E)** Bar graph depicts the frequency distribution of social caste. [BW, Backward; SC, Scheduled Caste] **(F)** Bar graphs elucidating the prevalence rates of different psychiatric disorders, notably emphasizing a heightened occurrence of alcohol dependence.

In the present study, a total of 1,016 individuals were included, with females constituting the majority (n=542, 53.35%) and males making up the rest (n=474, 46.65%). As detailed in **Table 1**, the age distribution shows that the largest group is young adults aged 18-30 (42.91%) (**Figure 2A**), with a higher proportion of females (46.86%) compared to males (38.40%). The middle-income group is the most represented socioeconomic status (46.45%) (**Figure 2B**), and the Scheduled Caste (SC) group constitutes the majority in terms of social caste (56.98%) (**Figure 2E**). It is important to note that in the Indian context, the caste system includes a range of categories with varying social and economic statuses. SC are historically marginalized and positioned at the lower end of the social hierarchy. Conversely, there are higher caste groups, such as the Brahmins and Kshatriyas, who traditionally occupy higher social and economic positions (14). The high representation of SC individuals in our sample underscores the prevalence of socioeconomically disadvantaged populations in the study area and provides insight into the broader context of caste-based disparities. Marital status analysis reveals that 69.19% of individuals are married (**Figure 2C**), with a slightly higher percentage among females (71.40%). Educational status indicates that a significant portion of the population has a middle-level education (31.98%) (**Figure 2D**), with females showing a higher frequency of illiteracy (19.37%) compared to males (10.97%).

**Table 1:** Demographic Characteristics of Study Participants.

Variable	Grouping	Overall (n) (%)	Male (n) (%)	Female (n) (%)
**Sample Size**	NA	1016	474	542
**Age Group**	18 - 30 (Young Adult)	436 (42.91)	182 (38.40)	254 (46.86)
	31 - 43 (Early Middle Age)	315 (31)	161 (33.97)	154 (28.41)
	44 - 56 (Late Middle Age)	188 (18.50)	87 (18.35)	101 (18.63)
	57 - 60 (Transitional Phase to Old Age)	77 (7.57)	44 (9.28)	33 (6.09)
**Socio-Economic**	Upper Middle	118 (11.61)	62 (13.08)	56 (10.33)
	Middle	472 (46.45)	221 (46.62)	251 (46.31)
	Lower Middle	316 (31.10)	147 (31.01)	169 (31.18)
	Lower Class	98 (9.64)	39 (8.23)	59 (10.89)
	BPL	12 (1.18)	5 (1.05)	7 (1.29)
**Social Caste**	Open	307 (30.21)	152 (32.07)	155 (28.60)
	Backward	130 (12.79)	64 (13.50)	66 (12.18)
	SC	579 (56.98)	258 (54.43)	321 (59.23)
**Marital Status**	Married	703(69.19)	316 (66.66)	387 (71.40)
	Unmarried	272 (26.77)	151 (31.86)	121 (22.32)
	Widow/Divorce	41 (4.03)	7 (1.48)	34 (6.27)
**Educational Status**	Illiterate	157 (15.45)	52 (10.97)	105 (19.37)
	Primary (1^st^ - 5^th^)	32 (3.14)	12 (2.53)	20 (3.69)
	Middle (6^th^ - 8^th^)	325 (31.98)	159 (33.54)	166 (30.63)
	SSC (9^th^ - 10^th^)	305 (30.01)	156 (32.91)	149 (27.49)
	HSC (11^th^ - 12^th)^)	128 (12.59)	65 (13.71)	63 (11.62)
	Graduation (12^th^ - 15^th^)	69 (6.79)	30 (6.33)	39 (7.20)

NA, Not Applicable; BPL, Below Poverty Line; SC, Scheduled Caste; SSC, Secondary School; HSC, Higher Secondary School.

It was found that alcohol dependence emerges as the most prevalent overall, affecting 12.30% of the sample. Interestingly, there are notable gender differences in the prevalence of certain disorders (**Table 2**). Generalized Anxiety Disorder (GAD) and depression appear to be more prevalent among females, with rates of 5.72% and 3.32%, respectively, compared to much lower rates among males (0.21% and 1.05%) (**Table 3**). Conversely, Substance Dependence/Abuse shows a higher prevalence among males (2.74%) compared to females (0%). Other disorders, such as Manic Disorder, Dysthymia, Agoraphobia, Social Phobia, Psychotic Disorder, Panic Disorders, and obsessive-compulsive disorder, exhibit lower prevalence rates overall (**Figure 2F**), with some variations between genders (**Table 2**).

**Table 2:** Prevalence of Psychiatric Disorders by Gender in a Population Study.

Disorder	Overall (n=1016)	% [95 CI]	Male (n=474)	% (95 CI)	Female (n=542)	% (95 CI)
**Alcohol dependence**	125	12.30 [10.28-14.32]	125	26.37 [22.40-30.34]	0	0
**Generalized Anxiety Disorder**	32	3.14 [2.07-4.22]	1	0.21 [-0.20-0.62]	31	5.72 [3.76-7.67]
**Depression**	23	2.26 [1.34-3.18]	5	1.05 [0.14-1.97]	18	3.32 [1.81-4.83]
**Substance Dependence/Abuse**	13	1.28 [0.59-1.97]	13	2.74 [1.27-4.21]	0	0
**Manic Disorder**	6	0.59 [0.12-1.06]	3	0.63 [-0.08-1.35]	3	0.55 [-0.07-1.18]
**Dysthymia**	3	0.30 [-0.04-0.63]	1	0.21 [-0.20-0.62]	2	0.37 [-0.14-0.88]
**Agoraphobia**	2	0.20 [-0.08-0.47]	2	0.42 [-0.16-1.01]	0	0
**Social Phobia**	2	0.20 [-0.08-0.47]	2	0.42 [-0.16-1.01]	0	0
**Psychotic Disorder**	2	0.20 [-0.08-0.47]	1	0.21 [-0.20-0.62]	1	0.18 [-0.18-0.55]
**Panic Disorders**	1	0.10 [-0.09-0.29]	0	0	1	0.18 [-0.18-0.55]
**Obsessive Compulsive Disorder**	1	0.10 [-0.09-0.29]	1	0.21 [-0.20-0.62]	0	0
**Total**	210	20.67 [18.18-23.16]	154	32.49 [28.27-36.71]	56	10.33 [7.77-12.89]

**Table 3:** Association Values (Odds Ratio with 95% Confidence Interval) of Demographic Variables and Overall Psychiatric Disorders.

	Grouping	Overall (n/total N)	% [95 CI]	OR [CI]	P-value	Male (n/total N)	% [95 CI]	OR [CI]	P-value	Female (n/total N)	% [95 CI]	OR [CI]	P-value
**Age**	**18-30**	45/436	10.32 [7.47-13.18]	**Ref.**		32/182	17.58 [12.05-23.11]	**Ref.**		13/254	5.12 [2.41-7.83]	**Ref.**	
	**31 -43**	95/315	30.16 [25.09-35.23]	3.75 [2.53-5.54]	<0.0001	72/161	44.72 [37.04-52.40]	3.79 [2.31-6.20]	<0.0001	23/154	14.94 [9.31-20.56]	3.30 1.62-6.74	=0.001
	**44 -56**	47/188	25 [18.81-31.19]	2.89 [1.84-4.55]	<0.0001	32/87	36.78 [26.65-46.91]	2.72 [1.52-4.86]	=0.0007	15/101	14.85 [7.92-21.79]	3.23 [1.47-7.07]	=0.003
	**57-60**	23/77	29.87 [19.65-40.09]	3.7 [2.07-6.59]	<0.0001	18/44	40.91 [26.38-55.44]	3.24 [1.59-6.61]	=0.0012	5/33	15.15 [2.92-27.38]	3.31 [1.09-9.97]	=0.033
**Marital Status**	**Married**	163/703	23.19 [20.07-26.31]	2.3 [1.55-3.54]	=0.0001	121/316	38.29 [32.93-43.65]	2.98 [1.84-4.81]	<0.0001	42/387	10.85 [7.75-13.95]	2.82 [1.09-7.30]	=0.032
	**Unmarried**	31/272	11.40 [7.62-15.17]	**Ref.**		26/151	17.22 [11.20-23.24]	**Ref.**		5/121	4.13 [0.59-7.68]	**Ref.**	
	**Widow/Divorce**	16/41	39.02 [24.09-53.96]	4.9 [2.39-10.33]	<0.0001	7/7	100	71.03 [3.93-1282]	=0.0039	9/34	26.47 [11.64-41.30]	8.35 [2.57-27.06]	=0.0004
**Socio-Economic**	**Upper Middle**	12/118	10.17 [4.72-15.62]	**Ref.**		10/62	16.13 [6.97-25.28]	**Ref.**		2/56	3.57 [-1.29-8.43]	**Ref.**	
	**Middle**	98/472	20.76 [17.10-24.42]	2.3 [1.22-4.3]	=0.0098	70/221	31.67 [25.54-37.81]	2.41 [1.15-5.02]	=0.018	28/251	11.16 [7.26-15.05]	3.39 [0.79-14.6]	=0.102
	**Lower Middle**	60/316	18.99 [14.66-23.31]	2.02 [1.07-4.02]	=0.03	46/147	31.29 [23.80-38.79]	2.36 [1.10-5.07]	=0.026	14/169	8.28 [4.13-12.44]	2.43 [0.53-11.07]	=0.24
	**Lower Class**	36/98	36.73 [27.19-46.28]	5.1 [2.4-10.5]	<0.0001	25/39	64.10 [49.05-79.16]	9.28 [3.62-23.8]	<0.0001	11/59	18.64 [[8.71-28.58]	6.18 [1.30-29.3]	=0.021
	**BPL**	4/12	33.33 [6.66-60.01]	4.41 [1.15-16.87]	=0.029	3/5	60 [17.06-102.9]	7.80 [1.1-52.82]	=0.035	1/7	14.29 [-11.64-40.21]	4.50 [0.35-57.3]	=0.24
**Educational Status**	**Illiterate**	41/157	26.11 [19.24-32.99]	7.77 [2.31-26.09]	=0.0009	24/52	46 [32.60-59.70]	7.71 [2.07-28.6]	=0.0023	17/105	16.19 [9.14-23.24]	15.62 [0.91-266.3]	=0.057
	**Primary**	8/32	25 [10-40]	7.33 [1.79-29.94]	=0.005	5/12	41.67 [13.77-69.56]	6.42 [1.22-33.6]	=0.027	3/20	15.00 [-0.65-30.65]	15.80 [0.77-322.5]	=0.072
	**Middle**	84/325	25.85 [21.09-30.61]	7.66 [2.34-25.03]	=0.0007	62/159	38.99 [31.41-46.57]	5.75 [1.67-19.77]	=0.005	22/166	13.25 [8.09-18.41]	12.30 [0.72-27.3]	=0.081
	**SSC**	60/305	19.67 [15.21-24.13]	5.38 [1.63-17.7]	=0.0056	48/156	30.77 [23.53-38.01]	4.00 [1.15-13.82]	=0.028	12/149	8.05 [3.68-12.42]	7.18 [0.41-124.0]	=0.17
	**HSC**	14/128	10.94 [5.53-16.34]	2.70 [0.74-9.74]	=0.129	12/65	18.46 [9.03-27.89]	2.03 [0.52-7.83]	=0.30	2/63	3.17 [-1.15-7.50]	3.21 [0.15-68.66]	=0.455
	**Graduation**	3/69	4.35 [-0.46-9.16]	**Ref.**		3/30	10.00 [-0.74-20.74]	**Ref.**		0/39	0	**Ref.**	

BPL, Below Poverty Line; SSC, Secondary School; HSC, Higher Secondary School; Ref, Reference.

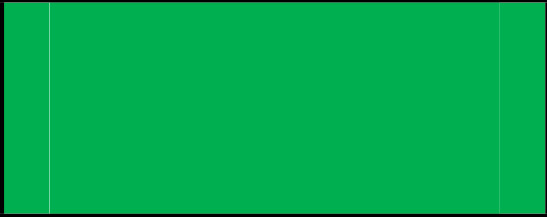
P value <0.05 (Significant Finding).

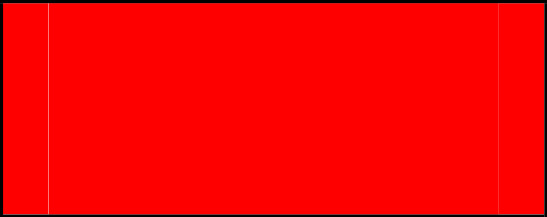
P Value >0.05 (Non Significant Finding).

**Figure 3:**
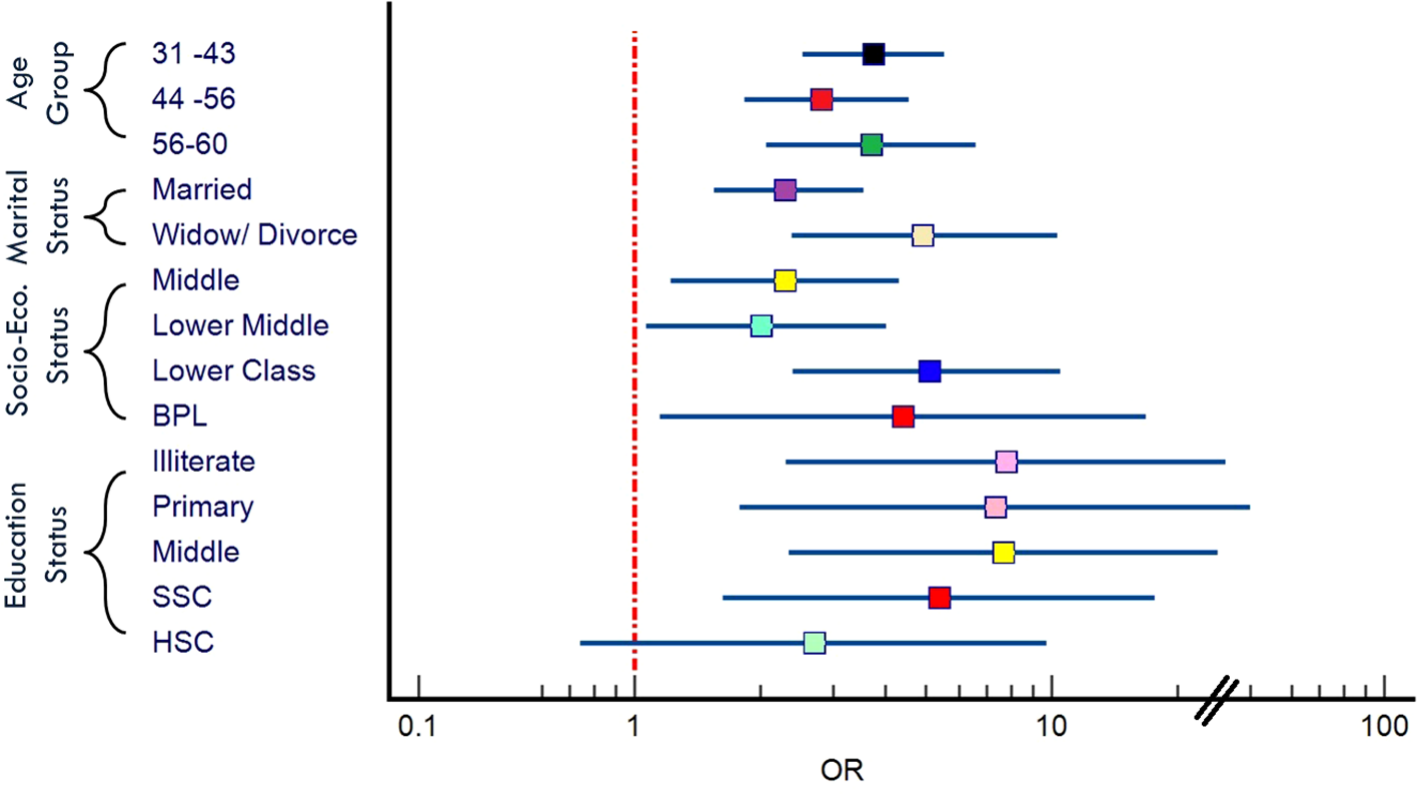
Forest plot delineating the association between various socio-demographic variables and the overall prevalence of psychiatric disorders [BPL, Below Poverty Line; SSC, Secondary School; HSC, Higher Secondary School].

**Figure 4:**
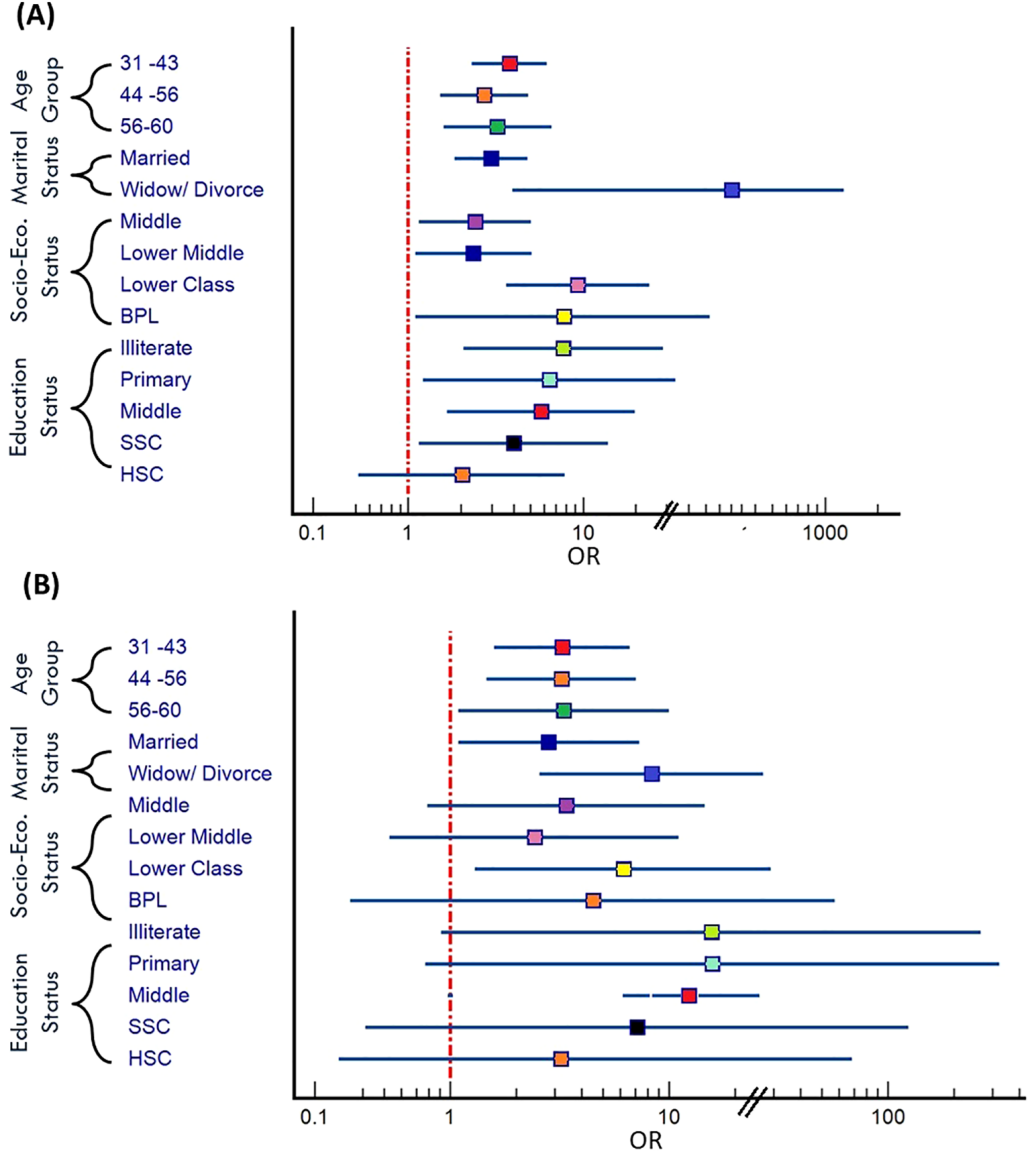
**(A)** Forest plot depicts the association between various factors and likelihood of psychiatric disorder in male **(B)** Forest plot depicts the association between various factors and likelihood of psychiatric disorder in female [BPL, Below Poverty Line; SSC, Secondary School; HSC, Higher Secondary School].

In our analysis, we also examined the association between various socio-demographic variables and the likelihood of overall psychiatric disorders (**Figure 3**). Firstly, concerning age groups, we noted significant disparities in the odds of psychiatric disorders across different age brackets. For males, compared to the reference group aged 18-30, the odds of experiencing psychiatric disorder were 3.79 times higher in the age group 31-43 (OR: 3.79 [2.31-6.20]), 2.8 times higher in the age group 44-56, and 3.7 times higher in the age group 56-60 (OR: 3.7 [2.07-6.59]) (**Figure 4A**). Similarly, for females, the odds ratios for these age groups compared to the reference were 3.25, 3.23, and 3.31, respectively (**Figure 4B**), indicating a noteworthy association between age and the likelihood of psychiatric disorders (**Table 3**). Married individuals showed 2.3 times higher odds of experiencing psychiatric disorder compared to unmarried counterparts, while the odds of experiencing psychiatric disorder were notably higher at 4.9 times (**Figure 3**) among those who were widowed or divorced compared to the unmarried group (**Table 3**). Thirdly, socio-economic status revealed a gradient effect, with lower socio-economic such as lower class OR: 5.1 [2.4-10.5] and Below Poverty Line (BPL) OR: 4.41 [1.15-16.87] classes showed progressively higher odds ratios (**Figure 3**) for psychiatric disorders compared to the upper middle class (**Table 3**). This suggests a clear association between socio-economic status and the likelihood of psychiatric disorders. Lastly, educational status displayed a similar trend, with decreasing educational attainment associated with higher odds of experiencing psychiatric disorders compared to the reference group with graduation-level education (**Figure 3**). Specifically, the odds ratios ranged from 5.38 to 7.77 across different educational categories, highlighting the significance of education in shaping susceptibility to psychiatric disorders (**Table 3**). However, we found several non-significant associations in the female group (**Figure 4B**).

In addition to socio-demographic variables, the study identified a significant number of individuals with psychiatric disorders who also showed chronic illnesses. These include hypertension (17.6%), diabetes (22.2%), infertility (84.6%), epilepsy (37.5%), and other factors such as disability and bereavement, as well as situations specific to families, such as having only female children (87.5%) (**Table 4**). It is important to note that the data related to disability, death, and only female children were relatively sparse. As a result, these events were grouped together and presented under the ‘Other’ category to ensure meaningful representation and avoid potential issues with statistical reliability due to small sample sizes.

**Table 4:** Distribution of Psychiatric Disorders by Presence of Chronic Illness/Stressful Events.

Chronic Illness/Stress Full events	No. Of People with Chronic Illness	No of people with psychiatric disorder	Frequency
Hypertension	17	3	17.6%
Diabetic	9	2	22.2%
Infertility	13	11	84.6%
Epileptic	8	3	37.5%
Divorce	41	16	39%
Other*	8	7	87.5%
Total	96	42	43.75%

*Others: disability, death and only female children in family.

## Discussion

The present study sheds light on the prevalence of psychiatric disorders among adults in rural Jammu and how socio-cultural and demographic factors might be linked to these disorders. By conducting a comprehensive survey involving 1016 individuals, the findings observed that psychiatric disorders affected a significant portion of the population, with an overall frequency of 20.67%. Notably, the prevalence rates were higher for males (32.49%), than for females (10.33%). Moreover, the survey highlights the widespread nature of alcohol dependence (**Figure 2F**). Furthermore, the investigation delineates varying rates of anxiety and depression across genders (**Table 2**).

In India, multiple studies have shown a high prevalence of psychiatric disorders. Sathyanarayana Rao and group reported a 24.40% prevalence in Karnataka, with significant issues such as depression, anxiety, and alcohol dependence (15). Similarly, Nair and group found a 33.9% prevalence of mental illness among people over 60 years old in rural Karnataka. It’s important to note that Nair et al.’s study specifically focused on this older age group, which differs from the age range in our study (16). In another study from the Kashmir region, Amin and Khan explored depression characteristics amid prolonged conflict, finding a high prevalence (55.72%), notably among 15 to 35-year-olds, with rural areas showing higher rates. Their research underscores the importance of culturally sensitive mental health initiatives to mitigate the conflict’s impact and improve overall well-being (9). A study conducted by Hussain and group found a point prevalence of mental illness in the Kashmir valley at 11.3% among the adult population, with a higher prevalence among females (12.9%) compared to males (8.4%). Depressive disorders were identified as the most common psychiatric condition (8.4%), followed by anxiety disorders (5.1%) (17). These studies not only reinforce the significance of our findings but also emphasize the urgent necessity for enhanced mental health services in rural areas. This underscores the critical importance of addressing mental health challenges in underserved communities and highlights the imperative for targeted interventions to ensure equitable access to care.

Our study focuses on the prevalence of psychiatric disorders in the RS Pura tehsil of the Jammu region, examining factors such as age, marital status, socio-economic status, and education level (**Figure 3**). We also identified significant stressors contributing to psychiatric disorders, including chronic illnesses like hypertension, diabetes, and epilepsy, as well as life stressors such as divorce and family-specific issues like having only female children (**Table 4**). Although our findings are specific to the RS Pura tehsil, similar patterns have been observed in other studies from different regions, which lends support to the relevance of these factors in rural settings (18–23). However, to avoid overgeneralization, we acknowledge that our results may not fully represent all rural areas. We suggest that further research is needed to explore whether the factors identified in RS Pura are applicable to other rural populations.

The findings from our study underscore the urgent need for enhanced mental health services in the rural area of Jammu region. There is a need to increase the availability of trained mental health professionals and to integrate mental health services into primary healthcare facilities. Community-based mental health centers, telemedicine, and mobile health clinics could significantly improve accessibility. Additionally, public education campaigns are essential to reduce stigma and encourage help-seeking behavior. Special attention should be given to supporting individuals with psychiatric disorders linked to chronic illnesses and significant life stressors. By working together with researchers, healthcare providers, policymakers, and community members we can make sure everyone has fair access to mental health care, no matter where they live.

### Strength, limitation, & future perspective

This study benefits from a well-defined sampling approach, utilizing a simple random sampling in the RS Pura region to ensure representative coverage of the rural Jammu region. The use of the MINI tool for psychiatric assessments provides a reliable and standardized diagnostic process, contributing to the robustness of the findings. Additionally, the study’s large sample size of 1016 participants allows for a detailed analysis of various socio-demographic factors and their associations with psychiatric disorders.

The study faces several limitations. The geographical focus on a specific rural area may limit the generalizability of the findings to other regions or urban settings. Additionally, the cross-sectional design restricts the ability to establish causal relationships between socio-demographic factors and psychiatric disorders. We also observed several non-significant associations within the female group, which may be attributed to the small sample size and variability within educational categories. To obtain more accurate and generalizable results, larger samples and more diverse data are needed.

Future research could benefit from broader sampling that includes diverse geographic locations and urban areas to improve generalizability. Longitudinal studies are valuable for understanding the progression and tracking changes in psychiatric disorders over time. Additionally, exploring other influencing factors such as cultural practices, healthcare access, and specific regional stressors could provide more comprehensive insights into mental health in the rural Jammu region.

## Conclusion

Mental health is vital to well-being, yet rural communities sometimes lack access to therapies and knowledge about psychiatric diseases. The present study sought to determine the prevalence of demographic characteristics linked with psychiatric diseases. Our investigation shows a complex link between socio-demographic factors and psychiatric problems. These findings help identify socio-economic gaps and demographic patterns in mental health outcomes, which is essential for establishing targeted interventions and policies to improve mental health.

## Data Availability

The raw data supporting the conclusions of this article will be made available by the authors, without undue reservation.

## Footnotes

1 [The random selection of villages was performed using the Excel function =RAND(), which generates random numbers to ensure an unbiased sampling process].
